# Analysis of the Knowledge, Attitudes, and Practice Model of Healthcare Professionals on Hearing Loss at Elderly Dementia Residences in Korea

**DOI:** 10.3390/healthcare10050792

**Published:** 2022-04-24

**Authors:** Chanbeom Kwak, Young Joon Seo, Kyoung Ho Park, Woojae Han

**Affiliations:** 1 Division of Speech Pathology and Audiology, College of Natural Sciences, Hallym University, Chuncheon 24252, Korea; cksqja654@gmail.com; 2 Laboratory of Hearing and Technology, Research Institute of Audiology and Speech Pathology, College of Natural Sciences, Hallym University, Chuncheon 24252, Korea; 3 Department of Otorhinolaryngology, Yonsei University Wonju College of Medicine, Wonju 26426, Korea; okas2000@yonsei.ac.kr; 4 Department of Otolaryngology-Head and Neck Surgery, College of Medicine, The Catholic University of Korea, Seoul 06591, Korea; khpent@catholic.ac.kr

**Keywords:** aging, hearing loss, dementia, KAP model, long-term care, healthcare

## Abstract

Due to a lack of knowledge about age-related hearing loss, its early identification and appropriate intervention are not being carried out in the field of dementia care. Since the untreated hearing loss of the elderly leads to a more rapid cognitive decline, the present study aimed to understand the hearing-related knowledge, attitudes, and practices of healthcare professionals in long-term care (LTC) facilities in Korea. A total of 557 workers (104 facility managers and 453 healthcare professionals) in residential LTC participated in this cross-sectional multicenter survey study. The Korean version of the knowledge, attitudes, and practice (KAP) on-line survey with a five-point scale or yes/no response was applied as the experimental tool. The results of structural equation modeling showed that knowledge significantly affected the attitudes and health-seeking practices of the facility manager, and allied healthcare professionals demonstrated similar results, which showed the significant effects of that knowledge on attitudes and health-seeking practices. This clearly indicated that sufficient knowledge is the driving force for the health-seeking practices and positive attitudes of both the facility manager and the healthcare professionals. Thus, we suggest that a further step, such as the development of comprehensive and professional guidelines regarding hearing care information for these professionals in residential LTC facilities, should be followed, and believe that this effort could lead to improving hearing-related knowledge, attitudes, and practices in order to clinically and politically care for the elderly population.

## 1. Introduction

Without a doubt, aging is the biggest risk factor for sensory impairments, which include hearing loss and dementia [1,2,3,4]. Age-related hearing loss (ARHL) is a representative disease caused by aging, prevalent among approximately 30% of people aged 65 to 74 years, and its probability increases by 40% to 60% with age for those 75 years or older [2,5]. The major problems caused by ARHL are poor communication ability and social isolation [2,3,6] and the possibility of deteriorating cognitive function [2,5], leading to dementia [3,4,7]. However, because hearing loss and dementia in the elderly share many common issues [8], it is easy to overlook the difficulties of hearing loss in dementia care and its management by both healthcare professionals and caregivers [3,9,10].

Although contemporary researchers have indicated that timeliness and appropriate methods for the intervention of hearing loss could alleviate the related symptoms for patients with dementia [2], most caregivers and/or allied healthcare professionals dealing with dementia have not been professionalized for hearing loss, especially in long-term care (LTC) facilities. Punch and colleagues systematically reviewed the impact of hearing loss and facility environment on residents in a LTC [7]. According to their review articles, healthcare professionals in an LTC facility unfortunately had limited knowledge for how best to care for hearing loss and carry out a qualified intervention and/or management of hearing aids for their patients with both hearing loss and dementia [7,11,12].

On the other hand, the knowledge, attitudes, and practices (KAP) model highlights the relation between the main components of knowledge (known), attitudes (believed), and practices (carried out) [13]. From the theoretical aspect, a lack of knowledge clearly influences healthcare professionals’ attitudes toward their patients and/or the environment and practice for health-seeking patients [14,15]. For example, when professionals have sufficient and technical knowledge, they have positive attitudes and active practices toward their patients, indicating that their attitudes are the catalyst of practices considered as an intention for behavior [13]. Taking advantage of this KAP model, several researchers applied it to the field of audiology to better understand health behavior and health-seeking practices, such as newborn hearing screening [16], the prevention of hearing loss [17], hearing conservation and/or preservation [18,19,20], and sensory impairments in those with dementia [6,21,22]. Recently, Dawes and his colleagues studied a cross-sectional multinational survey to identify the relation between three categories of the KAP model for the hearing and vision health of dementia patients in residential LTC, and compared these variables between nations [6]. They conducted a KAP survey of staff of LTC, which mainly addressed dementia patients with hearing and/or vision impairments in six countries, namely, England, South Korea, India, Greece, Indonesia, and Australia. The results of this international study revealed that most healthcare professionals in the LTC facility were aware that their dementia patients had hearing and/or vision impairments and needed screening tests and interventions by using assistive devices. However, the respondents had little knowledge about how to perform screening tests and/or interpret the results of these tests, manage or check the assistive devices, and use appropriate referral pathways. Clearly, differences exist in the uniqueness, specificity, and medical personnel between countries [6]. That is, the respondents in South Korea and Indonesia had relatively lower knowledge of the hearing and/or vision status of their patients than other countries, and also had less information regarding whether their patients wore assistive devices, e.g., hearing aids or spectacles. Furthermore, this lack of knowledge negatively influenced practices among the respondents of South Korea, especially for the testing or checking of hearing aids or spectacles [23], indirectly confirming that specialized education/training and the support of dementia patients with sensory impairments is needed.

Although this international cross-sectional study had advantages in terms of a comparison between nations with the same notion, there were several inevitable limitations, i.e., an unbalanced sample population [6]. In a similar way, the study by Dawes et al. [6] was conducted in six different nations, but it was an uncertain study since each study sample had representative elements stemming from the different systemic policies and infrastructures. Furthermore, the characteristics of LTC varied across the countries. Especially in South Korea, based on the rapid growth of the aged population and its importance, in 2010, the Ministry of Health and Welfare of Korea announced the First National Dementia Plan (NDP-1) to encourage the early detection of dementia and provide medical support for the aged population [24]. These efforts led to the establishment of the institutional LTC in Korea. The established system of LTC significantly changed the manner of care from a traditional family-based setting to a systematic and institutional community-based LTC. Each LTC center had an interdisciplinary team according to each specialty, such as counseling and registration, early detection of dementia, daycare service, family support, and dementia awareness and public information [24]. Even within Asian regions, most LTC in India still comprises a system of home-based care by family members. This discrepancy may be derived from the involvement of the Government. South Korea actively reformed and updated some policies and systems related to the LTC, whereas the LTC of India is not yet regulated or supported by the Government [6]. Thus, the representative data of each nation should be clearly verified. This effort may lead to establishing a basis for a highly evidenced-based international study. In light of this issue, the present study aimed to scrutinize the hearing-related knowledge, attitudes, and practices of those healthcare professionals who work for the elderly with dementia on a national level. Our hypotheses were as follows: (1) a higher amount and/or expansion of knowledge of the elderly with dementia and hearing loss positively affects views toward health-seeking practices; (2) a higher amount and/or expansion of knowledge of the elderly with dementia and hearing loss positively affects those attitudes; and (3) active attitudes toward the elderly with dementia and hearing loss positively affect health-seeking practices.

## 2. Materials and Methods

### 2.1. Study Design and Characteristics of Participants

**Figure 1:**
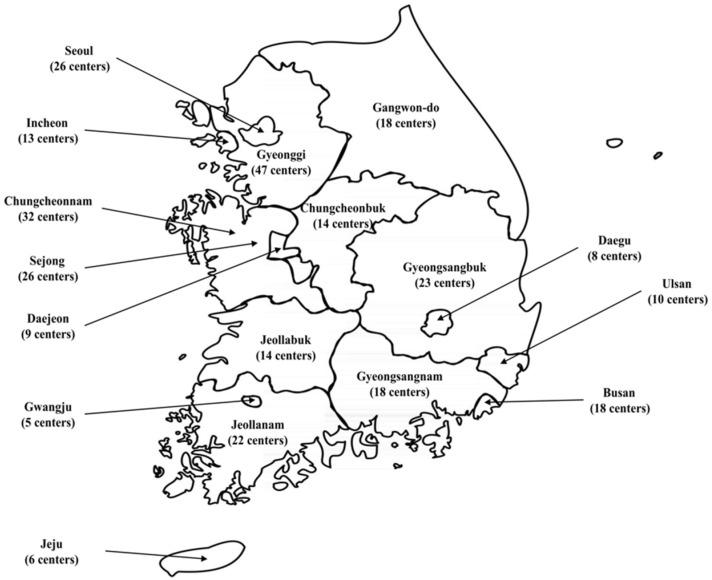
Graphical information on long-term care centers in the Korean nation.

A study design of a cross-sectional multicenter survey was adapted to investigate self-reported knowledge, attitudes, and practices of the healthcare professionals toward older adults with hearing loss. These healthcare professionals included clinicians, nurses, social welfare workers, care workers (i.e., care assistants, support workers, and nursing home assistants), and other allied healthcare professionals in the long-term care (LTC) facilities of South Korea. According to the Ministry of Health and Welfare and National Medical Center, there was a total of 286 LTC centers with approximately 3153 workers as of October 2020 [25]. In detail, 26 LTC centers were located in the Seoul Metropolitan City, and 66 centers were spread out in 7 cities (Incheon, Daejeon, Gwangju, Daegu, Ulsan, Busan, and Sejong). Additionally, 9 provinces (Gyeonggi, Gangwon, Chungcheonbuk, Chungcheonnam, Jeollabuk, Jeollanam, Gyeongsangbuk, Gyeongsangnam, and Jeju) had 191 LTC centers located in their cities. Figure 1 shows graphical information to help the reader understand the geography of Korea and the distribution of these centers.

To consider the equal distribution of participants and minimize any location bias, all the 286 LTC centers were selected and asked to enroll in the present study. The data collection was conducted between December 2020 and August 2021. Of the 3153 workers in these 286 LTC centers, 557 workers (104 facility manager and 453 healthcare professionals) participated in the on-line survey. The demographic information for these 557 workers is presented in Table 1. The section of job title shows the various job titles of the respondents. Although the participants were grouped by either facility manager or healthcare professionals regarding their current duty and position, there existed some duplications, which meant that one facility manager could be also a nurse.

All procedures of the current study were approved by the Institutional Review Board of Hallym University (Approval Number: #HIRB-2020-080) and all experiments were conducted in compliance with the Declaration of Helsinki, International Conference of Harmonization Guidelines for Good Clinical Practice. Before conducting any experiment, the participants had to agree to the consent form.

### 2.2. Survey Tool

Based on the knowledge, attitudes, and practice (KAP) theory, which is a theoretical model for human health behavior (homogeneous concept with practice), practice is affected by knowledge and attitudes of oneself [26]. To consider the characteristics of population and the objective of the study design, a modified Korean version of the KAP survey (K-KAP) was adapted [8]. The K-KAP survey was designated to identify the knowledge, attitudes, and practice factors of healthcare professionals who were related to elderly medical welfare facilities and the long-term hospital facilities. The target population for the K-KAP survey was specified in two versions: (1) facility managers and (2) doctor, nurses, and/or allied healthcare professionals. For the facility manager version of the questionnaire, 42 items are included, including demographic information (5 items), facility information (8 items, including 2 additional choice-dependent items), knowledge area (5 items, including 1 additional choice-dependent items), attitudes area (5 items), and practice area (19 items, including 7 additional choice-dependent items). In the K-KAP survey for doctor, nurses, and allied healthcare professionals, a total of 25 items are included, including 7 items of demographic information (1 additional choice-dependent item), 3 items of facility information, 7 items of knowledge area (1 additional choice-dependent item), 4 items of attitude areas (1 additional choice-dependent item), and 4 items of practice area (1 additional choice-dependent item). Excluding items related to demographic information and facility information, all items except for practice area (i.e., yes or no choices) were responded to on a 5-point Likert scale. Each version of the survey tool is depicted in Appendix A.

### 2.3. Main Outcome Measurement

In order to classify the items between facility manager and healthcare professionals, “M”, which stands for facility manager, was attached in front of the item number (i.e., MK1 implied #1 item of the knowledge area in the facility manager version). In the same way, “H” (representatives of healthcare professionals) was positioned in front of the item number in the healthcare professionals’ version.

For facility managers, in detail, the knowledge area asked questions such as the number and degree of hearing loss patients (MK1); knowledge and management of hearing test tools, including a screening test tool, questionnaire, and equipment (MK2); how hearing loss patients were referred to a clinician (MK3); and any audiological support in terms of facility management (MK4). All responses except for the practice area (yes or no choices or asking an open question to describe their thoughts) were quantified on a 5-point Likert scale (1: strongly disagree to 5: strongly agree). The mean score of the knowledge area was 1.12 (SD: 1.18) with a 0.76 reliability as measured by Cronbach’s alpha coefficient. The attitudes area was designated to measure the willingness and feasibility of their attitudes. These items included: the feasibility of a hearing test as a simple screening for their patients (MA1), willingness to seek clinical guidelines (MA2), necessity of having a routine hearing test (MA3), relationship between hearing loss and dementia (MA4), and necessity of having a hearing and cognitive test (MA5). The reliability of attitudes aspect was relatively high (Cronbach’s alpha: 0.74, mean: 2.57, SD: 1.02). With purpose of identifying the presence of the actual practice themselves, the practice area (mean: 3.01, SD: 0.48, Cronbach’s alpha: 0.37) included the following items: possibility (MP1) and feasibility of a hearing test (MP2), presence of clinical guidelines (MP3), specialists in hearing (MP4), education and support of hearing-assistive devices (MP5), hearing loss (MP6), and communication strategy (MP7), communication with patients (MP8) and communication with family members (MP9), providing support information for patients (MP10), and usefulness of information received from a hearing specialist (MP11).

The knowledge area (mean: 1.38, SD: 0.89, Cronbach’s alpha: 0.83) of healthcare professionals included an awareness of hearing loss patients (HK1) and hearing tests (HK2), expertise in the interpretation of hearing test results (HK3), a referral process for hearing loss patients (HK4), request for audiological support (HK5), and confidence of care of dementia patients with hearing loss (HK6). The unlikely knowledge area and attitudes area consisted of 3 items: the feasibility of a hearing test in both simple testing and screening for their patients (HA1), intention to seek clinical guidelines (HA2), and the effectiveness of hearing aids in patients (HA3). The mean score and reliability of attitudes areas were 2.12 (SD: 0.85) and 0.67, respectively. The practice area (mean: 3.07, SD: 0.26, Cronbach’s alpha: 0.37) intended to measure the actual practice of patients in their facility, for example, whether they conducted hearing aids management (HP1) or not, the presence of a hearing specialist (HP2), and the education history of hearing aids management (HP3).

### 2.4. Statistical Analysis

All variables were pre-processed for statistical analysis using R statistical computing software [27]. The structural equation model (SEM) was applied to identify the direct and/or indirect effects between the latent variable and the relationship between the latent variable and observed variable. The maximum likelihood estimate (MLE) method was used to confirm a parameter estimation. For goodness-of-fit measurement, absolute fit (i.e., significance of chi-square (*p* > 0.05), root mean square error of approximation (RMSEA), and goodness-of-fit index (GFI)), incremental fit (i.e., adjusted goodness-of-fit index (AGFI), comparative fit index (CFI), and normed fit index (NFI)), and parsimonious fit (i.e., chi-square/degree of freedom ratio (χ^2^/df)) were used. The statistical analysis process was conducted using the two-step approach, while indicating the validation of the pooled measurement model using a confirmatory factor analysis (CFA) as the first step and confirmation of the relationship between the variables in the structural model using path analysis as the second step [28].

## 3. Results

### 3.1. Descriptive Statistics and Self-Reported Knowledge, Attitudes, and Health-Seeking Practices

The information from the facility where the respondents to the present study were working is presented in the form of job-specific questions for the facility manager in Table 2 (number of workers and patients in the facility; proportion of patients with hearing loss, hearing aids, and dementia; and their gender-specific proportion and training of healthcare professionals, i.e., presence of dementia-relevant education). Especially for the questions related to percentage of the patients with dementia and hearing loss, the results were estimated based on the observation of the facility managers.

Additionally, some open questions were provided for both the facility manager and healthcare professionals to identify the specific reasons for their responses. In the knowledge area, the healthcare professionals were asked why they were not confident in treating patients with hearing loss who required hearing aids. The major reason for this was a lack of education on the use and/or maintenance (20.53%). The other reasons were a lack of education in checking and/or cleaning hearing aids (17.22%), turning hearing aids on/off (10.60%), checking to see whether hearing aids were working or not (4.42%), and changing the batteries of a hearing aid (1.32%). The healthcare professionals were also asked about attitudes, such as the reason why the patients did not use a hearing aid effectively. The reasons healthcare professionals gave were that the hearing aid was too expensive (28.70%), not appropriately fitted (13.02%), hard to use and not tolerated (11.26%), lost or broken (3.09%), and ineffective (1.99%). For health-seeking practices, the facility managers were asked to respond regarding the specific methods used to conduct the hearing (screening) test, the name of the test materials (i.e., questionnaire or test tool), the detailed role of designated workers who were responsible for hearing care, the detailed content of hearing education or communication strategies, and methods to help or communicate to patients with dementia and hearing loss. The facility managers responded that the reports of patients and family members were the most frequently used methods for hearing (screening) tests. They usually used a voice amplifier as the test and/or communication tools with patients. The alternatives included hearing aid counseling, referral to hospital, speechreading, and writing. Meanwhile, the healthcare professionals also responded regarding health-seeking practices, such as the testing or checking method in their practices. They conducted their practices based on the reports of patients (36.42%), checking whether the hearing aid was working or not (33.33%), and reports from family members (30.25%).

### 3.2. Validation of the Pooled Measurement Model

The results of the goodness-of-fit measurement for the basic model of facility manager revealed that it had several weak points in goodness-of-fit index, such as the *p*-value of chi-square < 0.001, 0.064 of RMSEA, 0.824 of GFI, 0.778 of AGFI, and 0.567 of NFI (see Table 3). In the case of healthcare professionals, the results of the goodness-of-fit measurement for the basic model are shown in Table 4. The basic model of healthcare professionals had ideal model fit indices, except for the significance of chi-square (*p* < 0.001) and 0.889 of the NFI value. To consider the modification indices for factor loadings, several modifications of the model were conducted, and the final model was thus derived.

Based on the results of the basic model of a facility manager, the covariant relations were established and corrected. After a repeated adjustment and model fitting, the final KAP model of the facility manager showed better fit indices than the basic model (χ^2^(24) = 119.36, *p* < 0.001). In addition, comparative results of the model indices between the basic and final model of healthcare professionals demonstrated that the final model had a greater statistically significant improvement than the basic model (χ^2^(24) = 151.68, *p* < 0.001). The results of the goodness-of-fit measurement for both the basic and final models for both groups are shown in Table 5.

### 3.3. Confirmation of Structural Model

**Figure 2:**
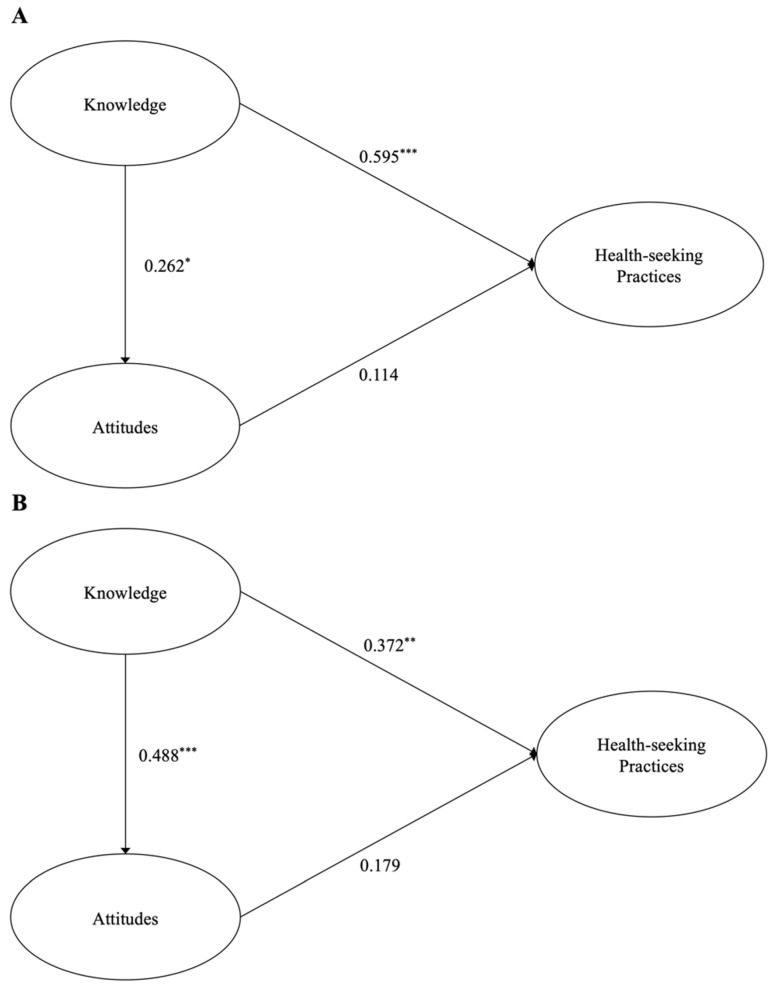
Results of the structural model using a path analysis for facility manager (**A**) and healthcare professionals (**B**). Asterisk indicates a statistical significance; ***: *p* < 0.001, **: *p* < 0.01, *: *p* < 0.05.

The structural model of the facility manager is depicted in Figure 2A. A path analysis was performed to identify the direct effect between the latent variables. Knowledge significantly and directly affected attitudes (regression coefficients: 0.262, *p* < 0.05) and health-seeking practices (regression coefficients: 0.595, *p* < 0.001). However, the direct effect of attitudes on health-seeking practices was not significant (regression coefficients: 0.114, *p* = 0.359). Similar to the results of facility manager, the path analysis of healthcare professionals demonstrated that significantly direct effects of knowledge were found (Figure 2B). In other words, two paths of knowledge were connected to attitudes (regression coefficients: 0.448, *p* < 0.001) and health-seeking practices (regression coefficients: 0.372, *p* < 0.01). Yet, unexpectedly, the direct effect of attitudes on health-seeking practices was not significant (regression coefficients: 0.179, *p* = 0.124). These results suggested that when both the facility manager and healthcare professionals expanded their knowledge, their attitudes positively affected and, consequently, led to better health-seeking practices and/or behavioral intentions to deliver those practices.

## 4. Discussion

The current study analyzed the associations between knowledge, attitudes, and the practices of allied healthcare professionals in the LTC facility on hearing loss who worked for the elderly with dementia using the SEM technique.

### 4.1. Impact of Knowledge on Attitudes and Health-Seeking Practices

The results from this current cross-sectional multicenter study suggested that both the facility manager and healthcare professionals had a limited and low level of hearing-related knowledge and attitudes toward their patients with dementia. As expected, these results were consistent with the cross-sectional multinational survey study of Dawes et al. [6]. They reported on the capacity of LTC staff who identify and manage hearing impairments in people with dementia and showed that Australians, Indonesians, and Koreans had a more negative awareness of their patients than those in other countries. For example, in the main outcomes of the KAP model, Koreans and Indonesians had limited knowledge of the hearing status of their patients, and they were not even aware whether their patients used hearing aids or not. Moreover, the majority of Koreans, Australians, and English were not exactly aware of the referral pathways or interdisciplinary care/managing and planning for dementia and hearing loss care [6]. The possible reason for this lack of knowledge and attitudes in the Korean LTC staffs may derive from insufficient training and/or education on hearing assessment/management. According to the Korean Health Insurance Policy Institute, more than half (56.3%) of the elderly in the LTC facility had a cognitive impairment and 20.3% of the elderly were diagnosed with dementia [6]. To care for and manage the elderly, the clinical practices related to the elderly were included in the training and education requirements. The majority of allied healthcare professionals in the LTC facility underwent these education and/or practical training. Unfortunately, the dementia professionals in the LTC facility were not sufficiently trained in hearing loss [23]. As mentioned in the Introduction section, Korea experienced a rapid change within several years from the existing patriarchal family-centered care for the elderly to a nuclear family and the expansion of health policies for the elderly. Therefore, it is expected that, in the next few years, as the elderly medical policy becomes well-stabilized, appropriate and sufficient professional education will be provided to LTC’s healthcare professionals. However, in spite of this lack of knowledge and attitudes for healthcare professionals that resulted in the absence of routine screening, uncertainty of conducting and interpreting assessments, and insufficient perspectives on interdisciplinary communication, the needs for actual clinical guidelines were highlighted [21].

The statistical analysis using an SEM technique in the current study revealed that knowledge is the driving force of health-seeking practices and attitudes for both the facility manager and healthcare professionals. Additionally, the results of facility managers demonstrated that hearing-related knowledge was more strongly associated with health-seeking practices than the attitudes toward their patients. However, the pattern of KAP model-based outcome measures differed amongst healthcare professionals. That is, greater knowledge of healthcare professionals was more strongly linked with attitudes of their patients than health-seeking practices.

### 4.2. Impact of Attitudes on Health-Seeking Practices

The descriptive data of our study showed that facility managers strongly agreed to all survey items related to attitudes (35.58% to 84.62%, which was higher percentage than for negative responses). However, the results for the same variable for healthcare professionals differed from the facility manager. For example, healthcare professionals negatively responded to the questions related to hearing aids (30.24% of respondents replied that they strongly disagreed and 19.65% strongly agreed). Thus, hearing-related knowledge and attitudes were limited for various reasons (i.e., inexperience in training or education of hearing aids), resulting in a strong association between the knowledge and attitudes of healthcare professionals. These results were supported by several studies with different academic fields [26,36]. In one of the previous studies by Hajj and colleagues, a similar methodological design to the present study was used for pharmacists to identify the major components of KAP theory and importance of educational needs in actual practices. They suggested that the lack of education and/or insufficient experience of professionals in their specific field could lead to low knowledge and attitudes [36]. Moreover, they emphasized the impact of interdisciplinary approaches for evidence-based healthcare services, and this effort could play a crucial role in the early screening, prevention, and intervention on healthcare professionals in LTC centers.

### 4.3. Limitation of the Current Study

This study has several limitations. One of the limitations is the limited variables for mediating effects in the KAP model. A poor communication ability in the patients with concurrent hearing loss and dementia was one of the main obstacles in clinical practice and a burden for allied healthcare professionals [3]. Mamo and colleagues developed a novel hearing care intervention for patients with dementia and analyzed it with various questionnaires, including the burden of the caregiver. They concluded that if the communication ability of dementia patients was boosted, the quality of life of these dementia patients and the burden of caregivers could be positively affected. When their results could not demonstrate statistically significant differences in the questionnaires for the time window of before and after interventions, a descriptive statistical analysis showed that the subjective burden of caregivers was reduced [3]. This finding suggests that the quantitative measurement of caregiver burden in the LTC facility may play an important role as a mediating variable. Thus, an adequate methodology and sophisticated analytic approach using various potential mediating variables is necessary to better understand patients with concurrent dementia and hearing loss and their caregivers in further research.

The other limitation was the lack of specific items on the survey instrument. Although the K-KAP survey used in the current study was derived from a part of the SENSE-Cog Program [21,37], which investigated the certain needs for individuals with dementia and sensory impairment, its fundamental focus was dementia-related sensory impairment such as hearing and vision. That is, the survey focused on identifying whether people with dementia had sensory impairments and, if so, whether healthcare professionals had the ability to manage them [37]. If the survey instrument was directly targeted toward age-related hearing loss and modified with hearing-centered items, more diverse and detailed analyses could be applied, and these analyses could be developed with more focus in future research.

## 5. Conclusions

The findings of the current study allowed us to explore the association between the main factors of the KAP model, analyzing them using sophisticated statistical methods and the SEM technique for allied healthcare professionals who care for and manage the elderly with dementia and hearing loss. First, the results showed that both the facility manager and healthcare professionals had a limited and low level of hearing-related knowledge and attitudes toward their patients with dementia. Secondly, the knowledge component was the driving force behind the attitudes and health-seeking practices of both the facility manager and healthcare professionals. However, the pattern of path analysis was different from the facility manager and healthcare professionals. The knowledge of the facility manager more positively impacted health-seeking practices rather than attitudes. On the other hand, knowledge towards attitudes was more positively and strongly affected in case of healthcare professionals. Based on the results of the study, we conclude that a comprehensive and professional hearing care guideline should be developed to improve hearing-related knowledge, change pessimistic attitudes, and motivate active practices as a positive further step.

## Figures and Tables

**Table 1:** Demographic information of enrolled participants (*n* = 557).

Variables	Facility Manager (*n* = 104)	Healthcare Professionals (*n* = 453)
Age (years, mean ± SD)	38.10 ± 12.73	38.51 ± 9.74
Gender (male: female)	7: 97	57: 396
Ethnic or Cultural Background (numbers, %)		
Korean	104 (100.00%)	452 (99.78%)
Others	0 (0.00%)	1 (0.22%)
Job Title (numbers, %)		
Facility Manager	5 (4.81%)	25 (5.52%)
Registered Nurse/ Licensed Practical Nurse	56 (53.85%)	213 (47.02%)
Nurse Aide/ Certified Nursing Assistant	0 (0.00%)	13 (2.87%)
Allied Healthcare Professional (inclusion of occupational therapists, physiotherapists, and other trained health care professionals)	2 (1.92%)	195 (43.05%)
Care Workers (care assistant, caregiver, etc.) (paid non-professional care workers, including front-line care workers and care assistants of patients with activities of daily living)	32 (30.77%)	7 (1.55%)
Others	9 (8.65%)	0 (0.00%)
Years in Profession (numbers, %)		
2 years or less	35 (33.65%)	72 (15.89%)
2 to 5 years	40 (38.46%)	69 (15.23%)
5 to 10 years	11 (10.58%)	165 (36.42%)
10 years or more	18 (17.31%)	141 (31.13%)
Professional Qualifications (numbers, %)		
Graduate school graduation	N/A	42 (9.27%)
University graduation	N/A	271 (59.82%)
College graduation (or equivalent)	N/A	119 (26.27%)
High school graduation	N/A	18 (3.97%)
Middle school graduation	N/A	3 (0.66%)
Facility Locations (numbers, %)		
Seoul	28 (26.92%)	50 (11.04%)
Incheon	5 (4.81%)	16 (3.53%)
Daejeon	1 (0.96%)	15 (3.31%)
Gwangju	1 (0.96%)	9 (1.99%)
Daegu	0 (0.00%)	6 (1.32%)
Ulsan	1 (0.96%)	0 (0.00%)
Busan	12 (11.54%)	46 (10.15%)
Sejong	0 (0.00%)	2 (0.44%)
Gyeonggi	10 (9.62%)	71 (15.67%)
Gangwon	4 (3.85%)	61 (13.47%)
Chungcheonbuk	7 (6.73%)	21 (4.64%)
Chungcheonnam	2 (1.92%)	41 (9.05%)
Jeollabuk	2 (1.92%)	15 (3.31%)
Jeollanam	5 (4.81%)	20 (4.42%)
Gyeongsangbuk	2 (1.92%)	24 (5.30%)
Gyeongsangnam	21 (20.19%)	48 (10.60%)
Jeju	0 (0.00%)	8 (1.77%)

Note: Values are expressed as a mean ± standard deviation for continuous variables and numbers with percentages noted for the categorical variables. Abbreviation: N/A: not applicable.

**Table 2:** Results of descriptive statistics of facility information responded by 104 facility managers.

Variables	Facility Manager (*n* = 104)
Number of workers (numbers, %)	
1 to 10	9 (8.65%)
11 to 20	77 (74.04%)
21 to 30	13 (12.5%)
31 to 40	4 (3.85%)
Over 40	1 (0.96%)
Number of patients (numbers, %)	
Under 1000	8 (33.33%)
1001 to 2000	7 (29.17%)
2001 to 3000	5 (20.83%)
Over 3000	4 (16.67%)
% of patients with dementia (numbers)	
0 to 25%	44.23% (46)
26 to 50%	25.96% (27)
51 to 75%	10.58% (11)
76 to 100%	19.23% (20)
% of patients with hearing loss (numbers)	
0 to 25%	48.08% (50)
26 to 50%	37.50% (39)
51 to 75%	12.50% (13)
76 to 100%	1.92% (2)
% of patients with hearing aids (numbers)	
0 to 25%	67.31% (70)
26 to 50%	28.85% (30)
51 to 75%	3.85% (4)
76 to 100%	0.00% (0)
Education Experience (numbers, %)	96 (92.05%)

Note: Values are expressed as a mean ± standard deviation for continuous variables and numbers with percentages for the categorical variables. The variable (number of patients) was the only item with fewer respondents (*n* = 24) than the other items (*n* = 104).

**Table 3:** Results of confirmatory factor analysis of facility managers to validate the pooled measurement model.

Latent Variable	Observed Variable	B	Beta	SE	AVE
Knowledge	MK1	0.693	0.621	0.186	0.621 ***
	MK2	0.716	0.664	0.132	0.664 ***
	MK3	0.384	0.447	0.160	0.447 *
	MK4	0.692	0.661	0.122	0.661 ***
Attitudes	MA1	0.372	0.379	0.126	0.379 **
	MA2	0.614	0.571	0.117	0.571 ***
	MA3	0.677	0.738	0.138	0.738 ***
	MA4	0.499	0.629	0.130	0.629 ***
	MA5	0.623	0.737	0.134	0.737 ***
Health-Seeking Practices	MP1	0.102	0.383	0.044	0.383 **
	MP2	−0.004	−0.032	0.004	−0.032
	MP3	0.055	0.220	0.034	0.220
	MP4	0.033	0.240	0.022	0.240 *
	MP5	−0.016	−0.097	0.015	−0.097
	MP6	−0.024	−0.127	0.012	−0.127 **
	MP7	−0.005	−0.024	0.011	−0.024
	MP8	0.230	0.696	0.047	0.696 ***
	MP9	0.271	0.615	0.048	0.615 ***
	MP10	0.287	0.611	0.039	0.611 ***
	MP12	0.145	0.175	0.094	0.175

Abbreviations: MK: knowledge of facility manager, MA: attitudes of facility manager, MP: practices of facility manager; asterisk indicates statistical significance; ***: *p* < 0.001, **: *p* < 0.01, *: *p* < 0.05.

**Table 4:** Comparison of results for goodness-of-fit indices for the basic and final models of healthcare professionals.

Latent Variable	Observed Variable	B	Beta	SE	AVE
Knowledge	HK1	0.448	0.484	0.049	0.484 ***
	HK2	0.616	0.755	0.054	0.755 ***
	HK3	0.645	0.776	0.040	0.776 ***
	HK4	0.602	0.771	0.038	0.771 ***
	HK5	0.569	0.793	0.035	0.793 ***
	HK6	0.454	0.540	0.040	0.540 ***
Attitudes	HA1	0.433	0.515	0.054	0.515 ***
	HA2	0.478	0.570	0.054	0.570 ***
	HA3	0.574	0.702	0.056	0.702 ***
Health-Seeking Practices	HP1	0.056	0.296	0.022	0.296 *
	HP2	0.142	0.526	0.031	0.526 ***
	HP3	0.096	0.308	0.024	0.308 ***

Abbreviations: AVE: average variance extracted, MK: knowledge of facility manager, MA: attitudes of facility manager, MP: practices of facility manager; asterisk indicates a statistical significance; ***: *p* < 0.001, *: *p* < 0.05.

**Table 5:** Comparison results of goodness-of-fit indices between the basic and final models for a facility manager and healthcare professionals.

Goodness-Of-Fit Indices	References	Facility Manager		Healthcare Professionals	
		Basic Model	Final Model	Basic Model	Final Model
Significance of chi-square	*p* > 0.05 Wheaton et al. [29]	0.000	0.922	0.000	0.857
RMSEA	RMSEA < 0.08 Browne and Cudeck [30]	0.064	0.000	0.072	0.000
GFI	GFI > 0.09 Jöreskog and Sörbom [31]	0.824	0.910	0.939	0.993
AGFI	AGFI > 0.09 Tanaka and Huba [32]	0.778	0.867	0.907	0.979
CFI	CFI > 0.09 Bentler [33]	0.901	1.000	0.919	1.000
NFI	NFI > 0.09 Bollen [34]	0.567	0.783	0.889	0.987
χ^2^/df ratio	χ^2^/df ratio < 3.0 Marsh and Hocevar [35]	1.432	0.837	3.35	0.72
		ANOVA results: χ^2^(24) = 119.36, *p* < 0.001		ANOVA results: χ^2^(24) = 151.68, *p* < 0.001	

Abbreviations: RMSEA: root mean square error of approximation, GFI: goodness-of-fit index, AGFI: adjusted goodness-of-fit index, CFI: comparative fit index, NFI: normed fit index, χ^2^/df: chi-square/degree of freedom ratio, ANOVA: analysis of variance.

**Figure A1:**
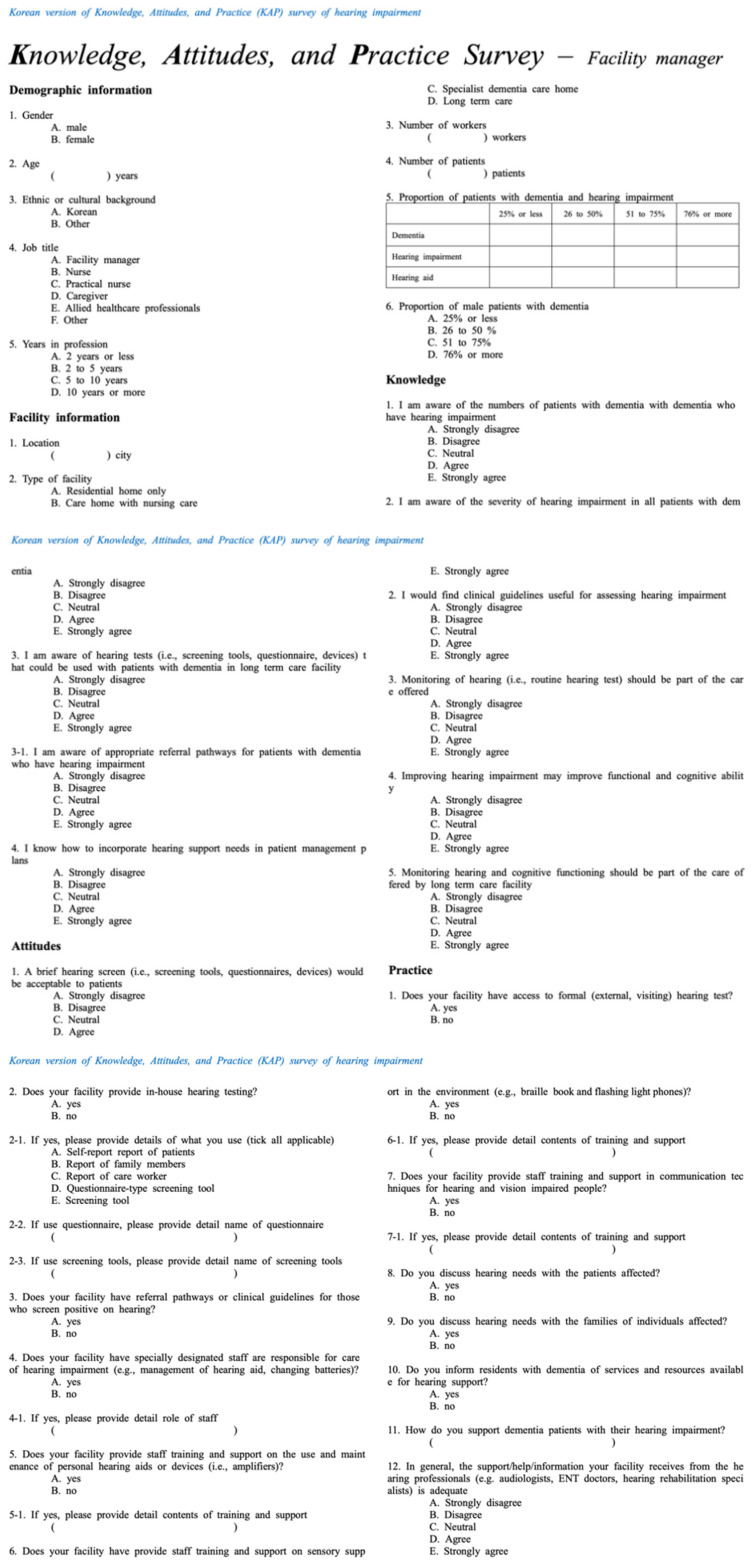
K-KAP survey for the facility manager.

**Figure A2:**
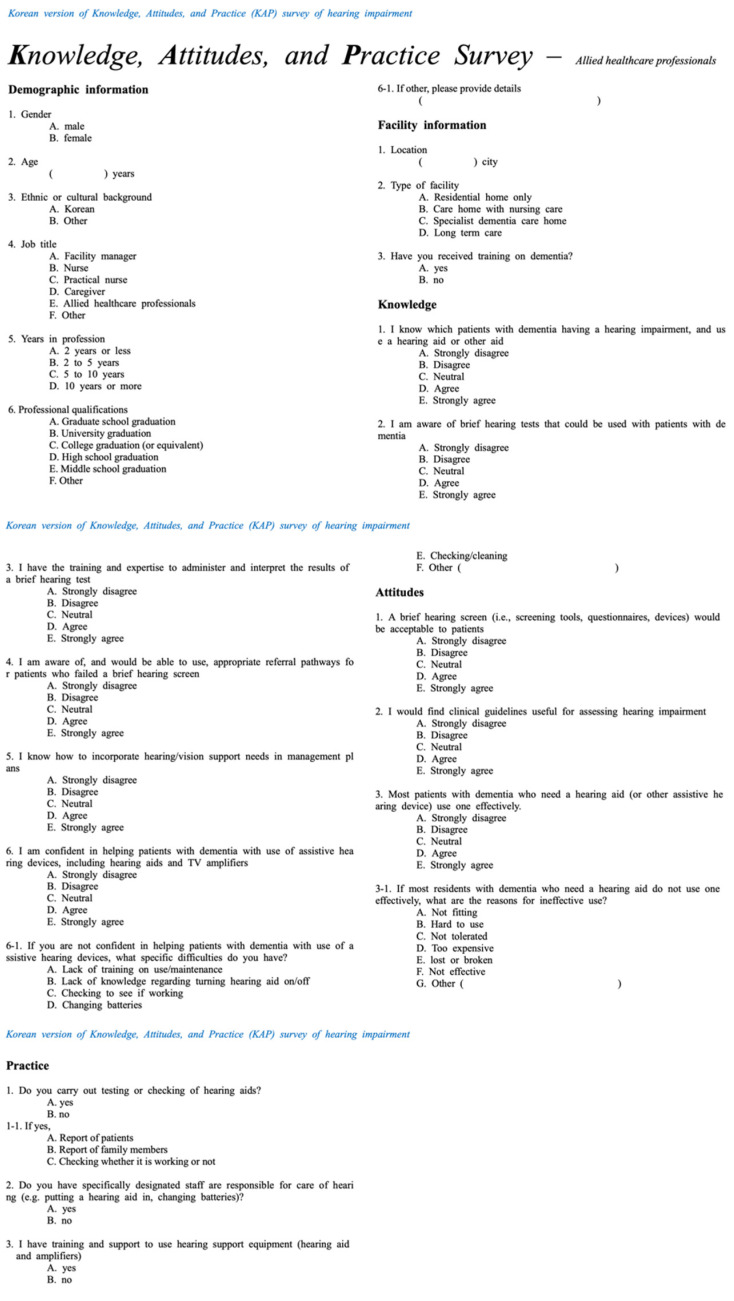
K-KAP survey for allied healthcare professionals.

## Data Availability

Not applicable.
